# Dipeptidyl Peptidase IV Inhibitory Peptides Generated in Dry-Cured Pork Loin during Aging and Gastrointestinal Digestion

**DOI:** 10.3390/nu14040770

**Published:** 2022-02-11

**Authors:** Paulina Kęska, Joanna Stadnik

**Affiliations:** Department of Animal Food Technology, Faculty of Food Science and Biotechnology, University of Life Sciences in Lublin, 20-704 Lublin, Poland; joanna.stadnik@up.lublin.pl

**Keywords:** dry-cured loin, LAB, bioactive peptides, dipeptidyl peptidase IV (DPP-IV)

## Abstract

The ability of peptides from an aqueous and salt-soluble protein extract of dry-cured pork loins to inhibit the action of dipeptidyl peptidase IV was determined. This activity was assessed at different times of the production process, i.e., 28, 90, 180, 270 and 360 days. The resistance of the biological property during the simulated digestive process was also assessed. For this, the extracts were hydrolyzed with pepsin and pancreatin as a simulated digestion step of the gastrointestinal tract and fractionated (>7 kDa) as an intestinal absorption step. The results indicate that dried-pork-loin peptides may have potential as functional food ingredients in the prevention and treatment of type 2 diabetes mellitus. In particular, the APPPPAEV, APPPPAEVH, KLPPLPL, RLPLLP, VATPPPPPPK, VPIPVPLPM and VPLPVPVPI sequences show promise as natural food compounds helpful in maintaining good health.

## 1. Introduction

Consumers’ nutritional awareness is growing, as is their interest in the importance of health-promoting components of the diet, such as biologically active peptides (BPs). BPs are inactive in the protein matrix, so they must be freed from sequence to show their positive or negative effects. They arise mainly where the hydrolytic decomposition of the peptide bond takes place by means of proteases. Therefore, these bioactive compounds are formed with the use of proteolytic enzymes (during digestion in the digestive system and their commercial counterparts in vitro), microbial fermentation (in which proteases of lactic acid bacteria are involved) or chemical hydrolysis. BPs have now been implemented in several measures, mainly as dietary supplements. Methods for their preparation include, but are not limited to, physical methods, such as membrane separation, nanofiltration and ultrafiltration, which are commercially available for the separation and purification of peptides on an industrial scale. However, as noted by Peighambardoust et al. [1], the costs of their use reach up to 70% of the total cost of production of BPs for food or nutraceutical applications, and, in many cases, this may affect the low profitability of industrial production of peptides. The solution may be to consume these BPs with foods in the daily diet. Food, especially fermented foods, such as yogurt and fermented meats, is a rich source of BPs [2,3,4]. In addition, BPs are exposed to proteases during digestion in the human digestive system, which may promote (but also lead to loss) their biological activity. It has been proven in the literature that the action of pepsin or pancreatin on dietary proteins has a positive effect on enhancing or stabilizing the biological activity of hydrolysates [4]. In addition, various factors, such as the type of food, processing conditions (e.g., meat long aging effect or cooking effect), protein source, amino acid sequence and composition, molecular weight and charge distribution, pH and some technological treatments, may directly affect the action of BP [5,6]. For this reason, more information is still needed to learn, for example, the relationship between the structure and activity of peptides, their resistance to external factors or possible interactions with other food components during digestion. Studies have shown that BPs from food can help prevent some of the most serious health risks, such as high blood pressure, obesity, atherosclerosis and diabetes [7,8]. That is why scientists provide new knowledge on the impact of BPs on the human body every year. In addition to the activity of BPs against oxidative factors, much attention is now paid to peptides that may contribute to lowering the risk of developing diabetes. Diabetes mellitus (DM) is a feature of a metabolic disease caused by elevated blood glucose levels due to insufficient insulin secretion or action, or both. DM can be of two types: (a) type I (insulin dependent) diabetes, which is an autoimmune disease in which beta cell dysfunction leads to little or no insulin being secreted by the pancreas; and (b) type 2 diabetes (T2DM), also known as non-insulin-dependent diabetes, where imbalances in insulin secretion and blood sugar absorption are observed [9]. T2DM accounts for as much as 90% of all cases. Other studies have shown that certain food-derived peptides can regulate sugar absorption and insulin levels in the body. One of their mechanisms of action is inhibition of dipeptidyl peptidase IV (DPP-IV). DPP-IV is a brush-membrane-associated prolyl dipeptidyl peptidase that is involved in the in vivo hydrolysis of incretins. DPP-IV is the enzyme responsible for the proteolytic breakdown of incretins, which play an important role in the regulation of glucose homeostasis. Incretins (intestinal insulinotropic hormones) are primarily responsible for regulating glucose levels. Two peptide incretin hormones involved in blood glucose control have been identified in humans, namely glucose-insulinotropic peptide (GIP) and glucagon-like peptide-1 (GLP-1). They are released from the gut in response to food intake and exert a strong insulinotropic effect (contribute to lowering glucose levels by stimulating insulin secretion and inhibiting glucagon release), helping to control postprandial glucose levels. However, incretins are quickly inactivated by the DPP-IV enzyme. Thus, blocking the action of DPP-IV increases the duration of action of incretins, which is one of the strategies for treating T2DM. So far, the presence of DPP-IV inhibitory (DPP-IVi) peptides in food products of animal origin has been demonstrated [10]. However, there seems to be no information available on potentially DPP-IVi peptides derived from proteins found in meat tissue during long aging.

The aim of this study was to understand the effect of production time on the formation of DPP-IVi peptides and to determine if these peptides are stable to simulated gastrointestinal digestion. According to our hypothesis, during the long-term aging of dry-cured pork loin, the profile of peptides changes, including those with DPP-IVi activity. Moreover, the use of selected lactic acid bacteria (LAB) strains intensifies these differences. In addition, protein fractions (water-soluble (WSF) and salt-soluble (SSF)) with the highest DPP-IVi properties in the in vitro test were subjected to spectrometric evaluation, and the obtained peptide profiles were screened in silico for the most promising dry-cured peptide sequences pork loins inoculated with LAB strains. The peptidomic profiles of in vitro digested meats were also determined to correlate possible differences in DPPi activity with the types and relative amounts of BPs released.

## 2. Materials and Methods

### 2.1. Dry-Cured Loins Preparation

Pork tenderloin (m. *Longissimus thoracis*) with an average weight of 2.0 ± 0.2 kg from carcasses of Great White Polish fattening was used for the research. At 48 h postmortem, all loins were dry-cured with a surface massage with a curing mix (2% sea salt, 0.97% curing salt and 0.03% NaNO_3_ per kg of tenderloin). After salting, the tenderloins were kept at 4 °C for 24 h to allow for the penetration of the curing mixture. After salting, part of the tenderloin was included (*n* = 3) as controls. The surface of the remaining tenderloins (*n* = 3 for each variant) was inoculated with 0.2% (*v*/*w*) probiotic strains to an initial level of 10^6^–10^7^ CFU/g of meat. The strains of lactic acid bacteria used are described in the literature in the production of cured meats. Their proteolytic potential was also determined, showing that they contributed to the formation of potential BPs. The pre-prepared loins were placed in a disinfected laboratory ripening chamber with a relative humidity of 75% ± 0.3 and a temperature of 16 ± 1 °C for 14 days. Then whole pieces of tenderloin were vacuum-packed in 80 µm polyamide (PA)/polyethylene (PE) bags (Wispak, Lublin, Poland) and aged at 4 ± 1 °C for 28, 90, 180, 270 and 360 days. Four independent experimental batches were conducted during these time periods.

### 2.2. Muscle Protein Extraction and Gastro-Intestinal Digestion

The water-soluble fraction (WSF) and the salt-soluble fraction (SSF) of the meat-muscle proteins were extracted on the basis of their solubility criteria, according to Molina and Toldra [11] and Fadd et al. [12]; as described in a previous study [13], WSF was obtained by homogenizing meat with cold distilled water in a ratio of 1:9 for 5 min (T25 Basic ULTRA-TURRAX; IKA, Staufen, Germany), followed by centrifugation (at 10,000× *g*, 4 °C for 20 min).The supernatants were collected.To prepare the salt-soluble fraction (SSF), the pellet resulting from the WSF extraction was re-suspended in 0.6 M NaCl in 0.1 M phosphate buffer (pH 6.2) in a ratio of 1:6 and homogenized for 5 min. The resulting homogenate was deaerated prior to extraction for 18 h at 4 °C. After the centrifugation step at 10,000× *g*, 4 °C for 20 min. The supernatants were filtered through Whatman Filter Paper No. 1. Protein concentration was determined by the Bradford method [14] and by using bovine serum albumin (BSA) as the standard (concentration range is 0–10 mg mL^−1^). Muscle protein fractions (WSF and SSF) were hydrolyzed in vitro by pepsin (2 h, E: S ratio 1:100 at pH 2.0) and then pancreatin (3 h, E:S ratio 1:50 at pH 7.0) at 37 °C, in the dark, and with constant stirring. After this, the enzyme was inactivated by heating at 95 °C for 10 min [15]. The peptides were then isolated from the hydrolysates by using dialysis (1:5, pH 7.4 phosphate buffered saline; <7 kDa molecular weight cutoff; Spectra/Por^®^ (Spectrum, Fort Worth, TX, USA)and under conditions of no light for 1 h at 37 °C) as a simulated intestinal absorption step and frozen at −80 °C for DPPi activity analysis.

### 2.3. Assessment of Peptides Content during the Digestion 

Protein hydrolysis after each step of in vitro digestion and simulated absorption was followed by measuring the amount of released primary amino groups, using the 2,4,6-trinitrobenzenesulfonic acid (TNBS) [16]. Leucine was used as a standard (witch concentration range is 0–5 mg mL^−1^).

### 2.4. Dipeptidyl-Peptidase IV Inhibitor Screening Activity Determination

A DPP-IV Inhibitor Screening Kit (MAK203, Merck, Burlingtown, MA, USA)was used for activity assay, and the procedure was carried out according to the manufacturer’s recommendations. Specifically, 50 μL of enzymes solution and 25 μL of sample solution were mixed and incubated at 37 °C for 10 min. Buffer, instead of enzyme solution, was used as a control. Then, 25 μL of DPP-IV substrate was added. The fluorescence (FLU, λex = 360/λem = 460 nm) was measured at 37 °C with a microplate reader (Varioscan Lux, Thermo Fisher, Waltham, MA, USA), once a minute in 30 min (T), and the relative DPP-IV inhibition (%) was calculated as follows:

Slope = (FLU2 − FLU1)/(T2 − T1) = FLU/minute

The DPP-IV inhibition assay was carried out by using 96-wellmicroplates by measuring fluorescence due to the release of 7-amido-4-methylcoumarin (AMC) by the action of DPP IV from the internally quenched fluorescent substrate Gly-Pro-7-amido-4-methylcoumarinhydrobromide (Gly-Pro-AMC).

### 2.5. Spectrometric Peptides (>7 kDa) Identification and Computational Study

Before spectrometric analysis, the peptide fractions were concentrated on a rotary evaporator, redissolved in 0.01 M HCl and purified by using a 0.45 µm filter. The analysis was carried out by using the liquid chromatography method coupled with tandem electrospray mass spectrometry (LC–MS/MS). Concentration and desalting of the samples was performed on the RP-C18 precolumn (Waters Corp., Milford, MA, USA), while the peptides were separated on the RP-C18 nano-Ultra Performance column (Waters), using a 180-min linear acetonitrile gradient (0–35%) and a flow rate of 250 nL min^−1^. The column outlet was directly connected to a mass spectrometer (Orbitrap Velos, Thermo Fisher Scientific Inc., Waltham, MA, USA). Mascot Distiller (version 2.4.2.0, Matrix Science Inc., Boston, MA, USA) was used to preprocess raw files. The obtained peptide masses and their fragmentation pattern were compared with the protein sequence database (UniProtKB, www.uniprot.org, accessed on 1 October 2021),using the Mascot search engine (Mascot Daemon v.2.4.0, Mascot Server v.2.4.1, Matrix Science, London, United Kingdom, Britain). “Mammals” was selected as the search criteria. The following search parameters were also used: enzymatic specificity, none; peptide mass tolerance, 5 ppm; fragment mass tolerance, 0.01 Da. Protein weight was left unlimited, and weight values were monoisotopic with a maximum of two skipped cuts allowed. Methylthiolation, oxidation and carbamidomethylationwere established as constant and variable modifications. Peptide sequences from unknown original proteins are not listed. Peptide identification was performed by using a Mascot search engine (Matrix Science) with a probability-based algorithm. An expected value threshold of 0.05 was used for the analysis (all peptide identifications had less than a 0.05% chance of a random match).

### 2.6. In Silico Prediction for Activity of the Identified Peptides

BIOPEP-UWM [17] was used to screen potentially bioactive fragments in the sequences of the identified peptides. The “Calculations” tool was used in the evaluation, and the search field was limited by defining the scope of the “dipeptidyl peptidase IV inhibitor”. Using this approach, the frequency of bioactive fragments occurrence in protein sequence (A parameter) was assessed and defined as the ratio of the number of fragments with DPP-IVi activity in a protein sequence and the number of amino acid residues of protein. The following computer tools were also used in the in silico analysis: CPPpred toolsfor prediction of cell penetrating peptides [18]; ToxinPred (online tool for protein and peptide toxicity prediction and hydrophobicity [19]; Aller Top 2.0 [20] for prediction of allergencity and iDPPIV-SCM (sequence-based predictor for identifying and analyzing dipeptidyl peptidase IV (DPP-IV) inhibitors peptides, using a scoring card method (SCM) [21].

### 2.7. Docking Study

The receptor used in performed docking studies is an A chain of DPP-IV protein (RCSB PDB no: 2QT9, at https://www.rcsb.org/structure/2QT9 (accessed on 1 October 2021). Prior to docking, the structure was cleared of any irrelevant bound structures. Cleaned structure used for the study was 2QT9_clean.pdb. Selected (seven) different peptide ligands were subjected to the docking study. Before the actual analysis, a prediction of ligands secondary structures was performed. The PEP2D method was used to predict peptides’ secondary structure. PEP2D implements models trained and tested on around 3100 peptide structures, and, in contrast to other known software packages, it was specifically trained to predict peptide structures, not proteins. It was observed to perform relatively better than PSIPRED in the prediction of sheets [22]. Peptide docking was performed by using CABS-dock standalone v0.9.18, a Python2.7 package that enables protein–peptide docking with backbone flexibility [23,24]. All analyses were performed with default CABS-dock settings. Additional assumptions include the following: (I) no knowledge about the binding site,(II) slight fluctuations of the backbone of protein receptor,(III) random initial peptide conformations and positions and(IV) no included reference complex PDB structure (for further RMSD calculations). The CABS-dock algorithm generates 10 identical copies of the ligand–receptor systems and performs a Replica Exchange Monte Carlo sampling method with random ligand positions within 20A from the protein structure. The simulations allow for full flexibility of the peptide ligand with slight fluctuations of the protein backbone. As a result, a set of 10,000 models are generated in 10 different trajectories. Subsequently, all models with unbound states are rejected, and remaining models are sorted by their lowest interaction energies. All filtered ligand–receptor systems are then clustered by using the k-medoid algorithm to select the final models.

### 2.8. Statistical Analysis

The experiment was realized in three repeats in each of the replications. The normality of distribution of the variables within groups was verified with the Shapiro–Wilk test, and Levene’s test was used to assess the equality of variances for avariable calculated for the groups. Data analysis was performed by two-way analysis of variance (ANOVA) at a significance level of *p* < 0.05. Multiple comparisons were performed by using Tukey’s post hoc test. All results are expressed as means ± standard deviation.

## 3. Results

The significance levels of the factors included in the experiment and obtained by the ANOVA are presented in Table 1. Treatment (inoculation), aging time and the interaction between them showed a significant effect on DPP-IV inhibiting activity (DPP-IVi) and proteolytic changes expressed as primary amino groups (-NH_2_).

### 3.1. Evaluation of the DPP-IVi of the Intact Proteins (Extracts) from Dry-Cured Pork Loins

Protein hydrolysis during the meat fermentation and aging process significantly affects the peptide profile and, thus, the degree/type of biological activity offered by the product. Moreover, the use of different microbial strains can generate specific sequences containing peptides with biological activity [13,25,26,27]. In this study, probiotic strains were used as starter cultures in the production of dry-cured pork loins, and their effect on the production of BPs with DPP-IV inhibitory activity during a 360-day production process was assessed.As shown in Table 2, protein extracts (WSF and SSF) showed a strong inhibitory effect on DPP-IV activity; the obtained values ranged from 61.01 to 73.70%for WSF and 68.98 to 84.97% for SSF. Moreover, the influence of the applied strain on the DPP-IV inhibitory activity of protein extracts from the products was observed. The use of LAB as a starter culture was associated with a decrease in the biological value of the LAB tests compared to the control in WSF after 28 days, and a significant increase in the DPPi activity of SSF in the batches with strain of LAB. With the aging period, the DPPi activity of the obtained extracts increased, reaching the most favorable values after 90 (WSF) and 180 (SSF) days, especially in the LOCK batches. After this time, a decrease in the DPP-IV inhibitory capacity on day 270 and 360 of the analysis in both fractions was observed. Interestingly, in the last research period (360 days), a lower DPP-IV inhibition capacity of extracts obtained from LAB products was noted than in the control samples.

### 3.2. Evaluation of the DPP-IV Inhibiting Activity of the Extracts during Simulated Gastro-Intestinal Digestion (SGID)

SGID of WSF and SSF extracts was performed to evaluate the stability of the peptides against gastrointestinal enzymes. As shown in Table 3, with respect to WSF, the relative DPP-IV inhibitory activity decreased on day 90 of aging and then increased with the continuous manufacturing process following pepsin treatment, regardless of the assay variant. A slightly different trend was observed for SSF (Table 4), where, in all analyzed variants, an increase in DPP-IVi activity was observed after 28 days, 90 days (except LOCK) and 270 days of aging after pepsin digestion compared to intact proteins. Interestingly, there was a decline in DPP-IV after 180 days under the SSF. Taking into account the effect of intestinal digestion in vitro, it was noted that, within WSF (Table 3), DPP-IV inhibitory activity decreased in the first (28 days) and the last (360 days) (except LOCK) period of the study. On the other hand, DPP-IVi activity remains at a similar (constant) level (except for LOCK) in the 180-day aging tests or increases after 270 days after pancreatin hydrolysis, regardless of the tested samples. Taking into account the effect of pancreatin in the hydrolysates in SSF, an increase in DPP-IVi activity was observed in the control sample (C), regardless of the duration of the dry-cured loins production process. In turn, a decrease in this activity was observed in LAB tests after 28, 90 and 360 days of aging, and an increase in DPP-IVi activity after 180 and 270 days. Thus, we can speculate that cleavage of the peptides by gastrointestinal proteases increases the inhibitory activity of DPP-IV, due to the release of more potent BPs, especially after 180 or 270 days in both fractions. Taking into account the last step of SGID, a decrease in the DPP-IVi activity of the hydrolysates was generally observed (>7 kDa) regardless of the analyzed fraction, and the greatest decrease occurred in the samples after 270 days of aging.

**Figure 1:**
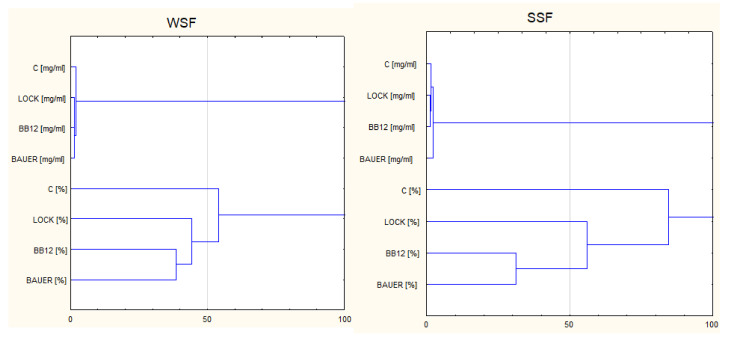
Dendrogram resulting from the Ward’s method of hierarchical cluster analysis of peptides (>7 kDa)concentration (mg/mL) and DPP-IV-inhibiting activity (%) in analyzed fractions WSF, water-soluble fraction; SSF, salt-soluble fraction; C, control sample; LOCK, sample inoculated with *Lactobacillus rhamnosus* LOCK900; BAUER, sample inoculated with *Lactobacillus acidophilus* Bauer Ł0938; BB12, sample inoculated with *Bifidobacterium animalis* ssp. *lactis* BB-12.

**Figure 2:**
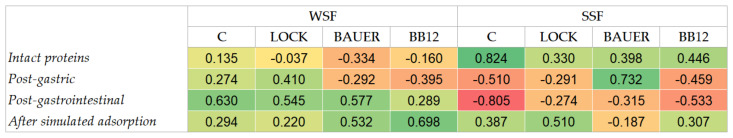
Correlation (Pearson’s correlation coefficient) between peptides content and DPP-IV-inhibiting activity of the tested samples. WSF, water-soluble fraction; SSF, salt-soluble fraction; C, control sample; LOCK, sample inoculated with *Lactobacillus rhamnosus* LOCK900; BAUER, sample inoculated with *Lactobacillus acidophilus* Bauer Ł0938; BB12, sample inoculated with *Bifidobacterium animalis* ssp. *lactis* BB-12.

Figure 1 and Figure 2 show the relationship between the content of peptides in the extracts/hydrolysates and the biological activity measured by the DPP-IV inhibition test. In general, the correlation between the factors under consideration has not been confirmed, and this prompted us to look for the properties of the peptides, apart from their quantitative index, which may affect the biological activity of specific sequences from the dry-cured pork loins. For this reason, the research was continued by focusing on specific peptide sequences in search of knowledge on potential DPP-IVi factors from meat products.

### 3.3. Peptide Profile of the Hydrolysates by LC–MS/MS and Computational Study

**Figure 3:**
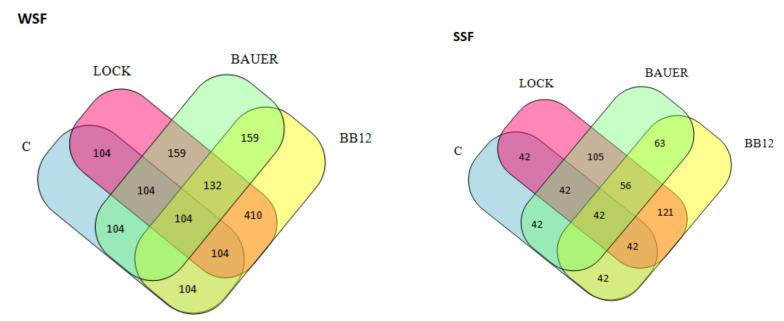
Venn diagram showing number of peptides obtained after SDIG of dry-curedpork loin after 180 days of ageing. WSF, water-soluble fraction; SSF, salt-soluble fraction; C, control sample; LOCK, sample inoculated with *Lactobacillus rhamnosus* LOCK900; BAUER, sample inoculated with *Lactobacillus acidophilus* Bauer Ł0938; BB12, sample inoculated with *Bifidobacterium animalis* ssp. *lactis* BB-12).

The obtained LC–MS/MS spectra made identifiable peptides characteristic of the LAB batches during the 12-month production period. Taking into account the value of (%) inhibition of DPP-IV and the trends observed during SDIG, we selected the six-month (180 days) aging period as the most favorable for both fractions. Therefore, the peptides (>7 kDa) obtained during the SGID after this time were subjected to further in silico analysis. During this time (180 days), a total of 1128 peptide sequences in WSF and 719 sequences in SSF were obtained, which occurred with different intensity in the analyzed variants. They also had a different peptide profile, depending on the fraction analyzed (WSF vs. SSF). A comparison of the unique and common (common) peptides identified in the analyzed variants, control and LAB, after 180 days are shown in Venn diagrams (Figure 3). The LOCK and BB12 batches were characterized by the highest common number of the same peptides, and this is consistent with the results of the HCA analysis. This observation well characterizes the ability of peptides to inhibit DPP-IV activity within SSF, where the mean inhibition value for LOCK and BB12 batches was 76.77% (*p* < 0.05) after SGID and was significantly higher (*p* > 0.05) compared to the remaining batches (mean value of DPP-IVi for C and BAUER batches was 65.82, *p* < 0.05). However, this trend was not confirmed within WSF, where the DPP-IVi value was as follows: BB12> C> BAUER> LOCK, and the differences between them were statistically significant (*p* > 0.05).

The spectrometric identification of the peptides allowed us to characterize the obtained fragments in order to ensure their functionality in terms of DPP-IVi. Peptide sequences after SGID (>7 kDa) from 180-day aging samples were analyzed in silico, assessing, inter alia, the frequency of bioactive (DPP-IVi) fragments in the protein sequence (parameter A), and selected peptides (with A greater than 1) are shown in Table 5.

**Figure 4:**
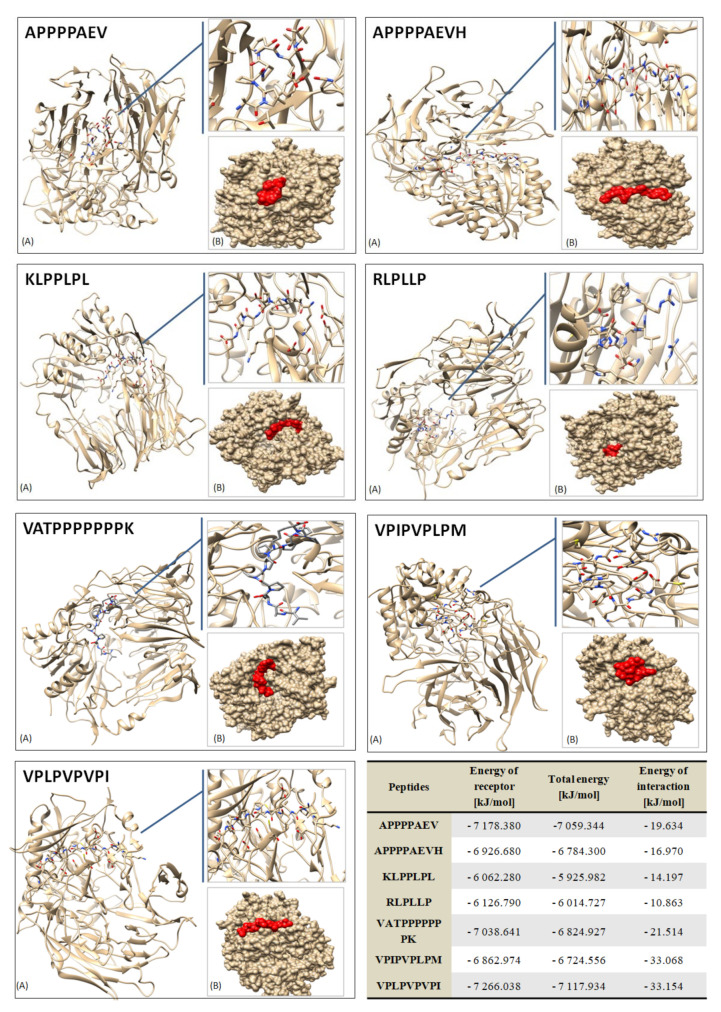
Three-dimensional molecular docking results of the receptor–ligand (peptide) systems in both surface representation (**A**) and cartoon-ribbon representation (**B**).

As shown by the analyses using the ToxinPred platform, the obtained peptide sequences from dry-cured pork loins showed no signs of toxicity. In contrast, 13 sequences out of 32 (i.e., 40%) of the peptides shown in Table 5 obtained the status “probably not allergen” and the other half obtained the status “probably not allergen (0)” by in silico analysis, using Aller Top 2.0. The bioactive properties of peptides are influenced by their amino acid composition, which, among the analyzed sequences, was as follows: among the peptides, Pro was most often identified, followed by Gly, Ala, Leu and, to a lesser extent, Val (Table 5). These amino acids belong to the group of polar (Gly) and non-polar (Pro, Ala and Leu), which predominantly shape the hydrophobic properties of peptides. In this study, the hydrophobicity ranged from −0.31 to 0.29 (Table 5). IDPPIV-SCM analysis shows that all identified peptides, except for GEAGPAGPAGPAGPR with LOCK and BB12, GDRGEAGPAGPAGPAGPR from BB12 batches, and AVSPGLAGPATK and SKRLPLP from the BAUER batches, were DPP-IV inhibitory peptides. Moreover, on the basis of the analyzed indicators, mainly DPP-IVi predictions, seven sequences particularly suspected of DPP-IV inhibition were selected and subjected to molecular docking analysis in order to check their possible existence as ligands of the DPP-IV protein molecule (2QT9; PDB). The 10 molecular-docking models were determined for each peptide. The most favorable model is shown in Figure 4.

## 4. Discussion

This study presents the potential of dry-cured pork loins as a source of peptides capable of inhibiting DPP-IV. This is a very important report, because peptides from food can become a good alternative to synthetic pharmacological compounds, especially taking into account their preventive action against lifestyle diseases, including diabetes [1,6,7,8]. There are several reports in the literature on peptides with antidiabetic activity in in vitro tests (DPP-IVi) from food [28,29,30], while relatively few of them have been devoted to meat or meat products. These were mainly in silico studies confirming the potential of meat for processing as a functional food, due to the presence of BP [25,31,32,33]. Fermented products, in particular, constitute an important group, due to these specific production processes in which peptides are released from the protein structure of the meat, promoting their bioactivity. It should be noted, however, that recently Martini et al. [34] found that pork (compared to beef, poultry and turkey) after ingestion and gastrointestinal digestion in vitro is the best source of DPP-IV inhibitory peptides. In addition, Marušić, Aristoy and Toldrá [35] analyzed the concentrations of natural peptides meat carnosine, anserine and GSH peptide in *B. femoris* muscle during long-term aging. GSH aged up to about 5 to 6 months and subsequently disappeared in the product, while carnosine and anserine were still present after 10 months of processing. Furthermore, these natural meat peptides were tested as DPP-IV inhibitors in in vitro studies, and the results showed that anserine and GSH did not inhibit DPP-IV activity at concentrations of 10 mM or less. However, carnosine had an inhibitory effect with an IC50 (half maximal inhibitory concentration) of 4.74 mM.Moreover, Gallego et al. [36] confirmed the presence of DPP-IV inhibitory peptides in the water-soluble extract of Spanish dry-cured ham, including carnosine; KA and AAATP; AA, GP, PL and AAAAG; ALGGA peptides; and LVSGM. This study showed the variability in the activity of peptides present in the extracts of pork loin at different aging periods (Table 2), caused by the variable activity of proteases (both from meat and microbial origin).Moreover, as we have already shown in our previous studies on antioxidant or angiotensin converting enzyme inhibitory activity, the biological activity of peptides also depends on their source, i.e., meat proteins that are either water-soluble (WSF) or salt-soluble (SSF) [13]. Taking both criteria into account, we see that the proteins extracted from the products show a strong DPP-IVi effect up to 180 days of aging (6 months), after which this activity decreases, possibly due to too intense hydrolysis, with WSF appearing to be more stable over the processing time.

When assessing food peptides as a factor supporting the preservation of good condition and human health, one should also take into account their resistance to hydrolysis during passage in the human gastrointestinal tract, where the consumed food is subjected to the action of, inter alia, proteases that break down proteins into peptides and amino acids. This can have a twofold effect: firstly, the bond in the structure of a very strong peptide may be broken down, causing it to lose its desired function; on the other hand, it may be released from the protein structure, increasing the potential of the hydrolysate to inhibit DPP-IV. Therefore, in this study, protein extracts from meat products were hydrolyzed with pepsin and pancreatin, thus reproducing the conditions in the human digestive system. The literature reports have shown the effect of this treatment on the action of peptides, i.e., an increase in DPP-IVi in whey and tuna derived peptides after digestion of the gastrointestinal tract in vitro [37,38]. Moreover, the boiled pork post-peptide fraction had a high DPP-IV inhibitory activity (IC50 = 1.88 ± 0.10 mg peptides/mL) compared to the intact extracts [34]. On the other hand, according to the research by Hardney et al. [39], no significant difference was recorded in the DPP-IV inhibitory activity for the three tested peptides, namely ILAP, LLAP and MAGVDHI, after simulated gastrointestinal digestion.

This study generally indicated a loss of capacity of the final hydrolysates compared to extracts, while they still showed DPP-IVi, although it was dependent on the enzyme used (loss of activity observed especially after two-stage hydrolysis with pepsin and pancreatin).By this approach, the presence of peptide DPP-IV inhibitors in digesta was confirmed, suggesting that these peptides can survive the gastrointestinal transit. It is also worth noting that the small peptides (two or three amino acids) formed during gastrointestinal digestion are more likely to be absorbed efficiently and reach the site of action along with the bloodstreamwithout losing their bioactivity. Although the size of the peptide does not disqualify the molecule as bioactive, as shown by Vilcacundo et al. [40], also high-molecular-weight quinoa peptides obtained by simulated gastrointestinal digestion, e.g., IQAEGGLT, a nine amino acid sequence, may be DPP-IV inhibitors.

In order to give meat peptides a “pro-health” status, they must also meet other criteria, including not being toxic or causing food allergies. The ToxinPred platform can predict toxic proteins and toxic peptides and identify toxic residues [18] and found that none of the peptides tested showed toxic potential. In contrast, several identified sequences are suspected of causing allergenicity. Although meat allergens do not constitute a large percentage of all food allergies, meat and its products cannot remain indifferent to allergy sufferers [19]. However, as noted by Goodman [41], in silico methods can determine whether a new protein is an existing allergen, or whether a new protein may cross-react with an existing allergen. However, they are not able to determine whether the new protein will “become” an allergen [41,42]. Moreover, as noted by Hayes et al. [42], the predictive value of seeking sequence similarity in terms of allergenicity potential should be carefully considered, as there is no perfect correlation between in silico results and in vivo food allergy occurrence. Thus, the level of potential allergenicity discovered in this study, or the absence of it, may not be indicative of allergy, but only to draw attention to a potential health problem. Further biological or biochemical or in vivo tests on sensitized individuals are necessary to confirm the prediction of allergy to peptides present in ripened meat products. Biologically active peptides must also have the ability to selectively penetrate cells to be used in biomedical health-related applications. These cellular penetrants work by traversing the cell membrane and delivering substances and macromolecules, showing higher delivery efficiency and less toxicity and immunogenicity problems than synthetic pharmacological substances with intracellular delivery [43,44]. Therefore, the “Probability of being cell penetrating” index determined, thanks to the in silico approach, was used as another criterion for the evaluation of peptides from meat. The study was based on CPPpred, which is a server for the prediction of cell penetrating peptides based on a novel N-to-1 neural network [20]. The study accepted the criterion that a result above 0.5 suggests that the peptide penetrates the cells. Results closer to 1 indicate that CPPpred is more confident that the peptide will penetrate cells, and results closer to 0 suggest that the peptide is very unlikely to penetrate cells. Admittedly, in this study, only three peptides met this criterion, i.e., KLPPLPL (0.550; C), SKRLPLP (0.610; BAUER) and RLPLLP (0.732; BAUER) (Table 5), but it may not be disqualifying for other peptide fragments when assessing their potential DPP-IVi. It should be noted, however, that, among them, two peptides (SKRLPLP and RLPLLP) have the status “probably allergen”. As explained above, this does not disqualify these sequences from further studies on the effects of DPPi, but points to the need for careful testing of these peptides for allergenicity in in vivo tests before they can be classified as health-promoting components of dry-cured pork loin.

It was noticed that it is not the amount of the peptides, but their specific properties resulting from their ammonium acid composition that cause their ability to inhibit the action of DPP-IV. For example, peptides made up of the amino acids Val (V), Thr (T), Arg (R), Gln (Q), Met (M), Leu (L), Lys (K), Phe (F) and Ala (A) are components of non-toxic peptides, while residues such as Pro (P), His (H)andCys (C) and the amino acid Asn(N) are commonly found in peptides exhibiting toxicity [45]. Although the identified sequences contained a lot of Pro in this study, the toxic effect was not predicted. On the other hand, studies have shown that peptides with good DPP-IV inhibitory activity in general have Pro and/or Hyp (O) amino acids in their sequence [39,46]. DPP-IV inhibitory activity may also be potentially due to the presence of an Ala residue in the penultimate position of the N-terminus. This is one of the structural features that appear to influence DPP-IV inhibitory activity. Moreover, the presence of hydrophobic amino acids—in this case, Met—at the N-terminal position is believed to increase the substrate specificity of DPP-IV [47]. Generally, it has been shown that peptides of 2–8 amino acids in which a Pro residue is on the second N-terminal position, and when this Pro residue is flanked on both sides by Val, isoleucine and/or Leu, it is most preferably flanked by Leu on one or both of them, and these sites act as good DPP-IV inhibitors [48]. The sequences selected for analysis and subjected to molecular refinement meet these criteria. The substrate specificity of DPP-IV is believed to be primarily based on the recognition of the positively charged N-terminus of the substrate by the Glu–Glu motif at the active site of the enzyme and the alignment of the amino acid residue at the P1 position into the hydrophobic pocket of the enzyme [49]. In this context, Pro and, to a lesser extent, Ala, particularly when in the penultimate position (P1), have a high affinity. This is supported by the argument that tripeptides diprotin A (IPI) and diprotin B (VPL), both containing a proline residue at the P1 position, are among the most potent peptides with DPP-IV inhibitory activity [46]. However, studies have shown that DPP-IV also cleaves peptides with Hyp, Ser, Gly, Val, Thr or Leu at this position [31,47]. In conclusion, the most potent peptides with DPP-IVi activity most preferably have Pro > Ala > Gly > Ser > Val > Leu at the P1 position [47], while the sequences found in this study mainly contained Pro > Gly > Ala > Leu> Val, which confirms the probability of their action in in vivo tests.

Of the many sequences identified in this study, seven sequences from pork loin after 180 days of maturation were selected as the best potential DPP-IV inhibitors; that is, molecular docking is used to virtually screen peptide sequences with bioactivities, including target proteins of interest. In this study, these sequences showed an affinity for the 2QT9 (PDB) molecule, and among them, the VPLPVPVPI peptide was the most energetically effective (it had the lowest interaction energy (kJ/mol)).It was a characteristic molecule for the test inoculated with *Lactobacillus rhamnosus* LOCK900.However, docking simulations alone cannot determine the differences between competitive and non-competitive binding interactions that affect the bioactivity of the peptide; therefore, the predicted bioactivity in this bioactivity study should also be analyzed in vivo.

The proteolytic activity of LAB is exerted in a strain-dependent manner, resulting in a wide variety of proteolytic activities [50]. Moreover, as noted by Jensen, Vogensen and Ardö [51], due to the specificity of the enzyme for the substrate, the peptide composition of the hydrolysates changes, and, thus, the activity of the peptides changes. In the previous study, we demonstrated the effect of the same LABs with different proteolytic activity on the intensity of protein transformations (based on the number of alpha-amino groups in the TNBS test) in pork loin [26]. This underlines that LAB can generate a large variety of BP, with varying activities, also as antioxidants [13] or ACE inhibitors [27]. We previously indicated that, regardless of the analyzed fraction (WSF or SSF) obtained from pork tenderloin after 180 days of maturation and subjected to hydrolysis, the most different results were obtained for products with the addition of the potentially probiotic *L. acidophilus* Bauer L0938 strain [13,27]. Protein hydrolysates obtained from the batches inoculated with this strain showed the strongest ACE inhibitory properties (SSF). On the contrary, within the SSF fraction, the samples marked as BAUER were characterized by the least favorable bioactive properties in the form of the ability to neutralize the ABTS radical. This indicates the need for the correct selection of strains that will be used in the production of dry-cured pork loins and, at the same time, contribute to the formation of BPs. In order to assess the influence of the applied strains on the biological activity of extracts and hydrolysates of proteins obtained from dry-cured products, a multi-dimensional method of cluster analysis was used, selecting the values of biological activity and the content of peptides as variables characterizing selected objects for the analysis. By using the cluster analysis method, the objects were divided into disjoint groups, indicating the largest or the smallest differences between the objects caused by the use of specific strains during the production of dry-cured loin. The influence of the strains used on DPP-IV and extracts and hydrolysates from the aging meat product was demonstrated. Thus, after 180 days of ripening of pork loins and in vitro hydrolysis, the strains used contributed to a higher DPP-IVi activity within SSF compared to the non-inoculated test (C). On the other hand, for the WSF fraction, the BB12 test showed the most favorable properties in terms of the considered bioactivity.

## Figures and Tables

**Table 1:** Significance levels shown by the experimental factors and their interactions for the DPP-IVi of dry-cured loins during long-term aging and in vitro gastrointestinal digestion.

Factor	Peptides Concentration (mg/mL)		DPP-IVi (%)	
	WSF	SSW	WSF	SSW
	*Intact proteins*			
Treatment (T)	N.S.	***	***	***
Storage time (S)	***	***	***	***
TxS	***	***	***	***
	*Post-gastric*			
Treatment (T)	*	N.S.	***	***
Storage time (S)	***	***	***	***
TxS	*	**	***	***
	*Post- gastrointestinal*			
Treatment (T)	***	N.S.	***	***
Storage time (S)	***	***	***	***
TxS	**	*	***	***
	*After simulated adsorption*			
Treatment (T)	N.S.	N.S.	***	***
Storage time (S)	***	*	***	***
TxS	N.S.	N.S.	***	***

NS, not significant; * *p* < 0.05; ** *p* < 0.01; *** *p* < 0.001. WSF, water-soluble fraction; SSF, salt-soluble fraction.DPP-IVi, dipeptidyl peptidase IV inhibiting activity

**Table 2:** Dipeptidyl peptidase IV inhibiting activity (DPP-IVi; %) and peptides concentration (PC, mg/mL) of intact proteins (extracts) of dry-cured pork loins.

Time (Day)		Variants							
		C		LOCK		BAUER		BB12	
		PC (mg/mL)	DPP-IVi(%)	PC (mg/mL)	DPP-IVi (%)	PC (mg/mL)	DPP-IVi(%)	PC (mg/mL)	DPP-IVi(%)
WSF	28	1.46 ^Ab^ ± 0.01	73.70 ^Ab^ ± 0.21	1.59 ^Ab^ ± 0.11	65.10 ^Cb^ ± 0.21	1.56 ^Ac^ ± 0.15	70.85 ^Bc^ ± 0.208	1.48 ^Ac^ ± 0.05	61.01 ^Db^ ± 1.63
	90	2.08 ^Ba^ ± 0.06	85.90 ^Ba^ ± 0.14	2.18 ^ABa^ ± 0.05	96.88 ^Aa^ ± 0.10	2.20 ^Aba^ ± 0.01	85.90 ^Ba^ ± 0.14	2.41 ^Ab^ ± 0.09	85.95 ^Ba^ ± 0.14
	180	1.69 ^Ab^ ± 0.13	84.51 ^Aa^ ± 4.31	1.35 ^ABc^ ± 0.15	68.89 ^Cb^ ±4.31	0.82 ^Cd^ ± 0.08	78.50 ^Bab^ ± 2.98	1.14 ^BCd^ ± 0.07	81.49 ^ABa^ ± 1.25
	270	1.42 ^Db^ ± 0.02	24.56 ^Bd^ ± 1.03	1.67 ^Cb^ ± 0.02	23.94 ^Bc^ ± 1.03	1.86 ^Bb^ ± 0.04	31.49 ^Ad^ ± 1.49	2.07 ^Aa^ ± 0.06	19.63 ^Cd^ ±0.80
	360	2.15 ^Aa^ ± 0.18	36.66 ^Ac^ ±2.22	2.14 ^Aa^ ± 0.03	14.99 ^Cd^ ± 3.66	2.04 ^Aab^ ± 0.14	27.73 ^Be^ ± 2.97	1.87 ^Aa^ ± 0.11	31.49 ^Cc^ ± 2.7
SSF	28	0.42 ^Bd^ ± 0.01	68.98 ^Cb^ ± 0.40	0.65 ^Ac^ ± 0.03	84.97 ^Aa^ ± 0.88	0.60 ^Ac^ ± 0.02	73.11 ^Bb^ ± 0.98	0.41 ^Bc^ ± 0.05	73.09 ^Bb^ ± 0.18
	90	1.21 ^Ba^ ± 0.04	54.80 ^Bc^ ± 0.33	0.93 ^Cb^ ± 0.03	51.38 ^Bb^ ± 3.78	1.15 ^Ba^ ± 0.04	64.21 ^Ac^ ± 3.62	1.34 ^Aa^ ± 0.03	61.32 ^Ac^ ± 0.22
	180	1.22 ^Aa^ ± 0.05	85.37 ^Aa^ ± 1.28	1.20 ^Aa^ ± 0.13	85.00 ^Aba^ ± 2.65	0.81 ^Bb^ ± 0.09	81.39 ^Ca^ ± 0.98	1.15 ^ABb^ ± 0.03	82.46 ^BCa^ ± 1.34
	270	0.56 ^Bc^ ± 0.001	15.73 ^Cd^ ± 3.61	0.45 ^Bd^ ± 0.06	30.63 ^Bc^ ± 2.20	0.79 ^Ab^ ± 0.02	34.04 ^ABd^ ± 2.44	0.52 ^Bd^ ± 0.03	36.01 ^Ad^ ± 0.21
	360	0.98 ^Ab^ ± 0.03	53.06 ^Ac^ ± 0.58	0.88 ^Ab^ ± 0.04	11.32 ^Cd^ ± 0.06	0.50 ^Bc^ ± 0.04	17.92 ^Be^ ± 2.59	0.56 ^Bd^ ± 0.01	10.71 ^Ce^ ± 0.52

WSF, water-soluble fraction; SSF, salt-soluble fraction; C, control sample; LOCK, sample inoculated with *Lactobacillus rhamnosus* LOCK900; BAUER, sample inoculated with *Lactobacillus acidophilus* Bauer Ł0938; BB12, sample inoculated with *Bifidobacterium animalis* ssp. *lactis* BB-12. All assays were performed in three replicates, and the results are presented as mean ± standard deviation; ^A–D^ Within the different treatment (verse), means followed by the commonletter do not differ significantly (*p* > 0.05).^a–e^ Within the different ageing time(column), means followed by the common letter do not differ significantly (*p* > 0.05).

**Table 3:** Dipeptidyl peptidase IV inhibiting activity (DPP-IVi; %) and peptides concentration (PC, mg/mL) of WSF protein hydrolysates during in vitro digestion of dry-cured pork loins.

Time (Day)		Dipeptidyl-Peptidase-IV-Inhibiting Activity(%)							
		C		LOCK		BAUER		BB12	
		PC (mg/mL)	DPP-IVi(%)	PC (mg/mL)	DPP-IVi(%)	PC (mg/mL)	DPP-IVi(%)	PC (mg/mL)	DPP-IVi(%)
Post-gastric	28	1.62 ^Ab^ ± 0.22	71.66 ^Ca^ ± 0.67	1.44 ^Ac^ ± 0.03	69.80 ^Cb^ ± 2.42	1.45 ^Ac^ ± 0.01	77.69 ^Bb^ ± 0.74	1.51 ^Ad^ ± 0.05	84.01 ^Ab^ ± 1.05
	90	1.51 ^Cc^ ± 0.02	48.23 ^Bd^ ± 3.12	1.54 ^Cbc^ ± 0.03	42.00 ^Bd^ ± 0.25	1.92 ^Ab^ ± 0.09	52.87 ^Ad^ ± 1.58	2.13 ^Abc^ ± 0.07	33.36 ^Ce^ ± 0.78
	180	2.05 ^Ab^ ± 0.14	84.51 ^Db^ ± 1.54	2.45 ^Aa^ ± 0.46	93.17 ^Ca^ ± 0.51	2.22 ^Ab^ ± 0.23	98.7 ^Aa^ ± 1.18	2.32 ^Aab^ ± 0.26	95.29 ^Ba^ ± 0.95
	270	1.94 ^Bb^ ± 0.04	64.370 ^ABc^ ± 0.834	2.00 ^Bab^ ± 0.03	60.33 ^Bc^ ±0.67	2.29 ^Aab^ ± 0.04	60.56 ^Bc^ ± 1.68	1.99 ^Bc^ ± 0.02	67.06 ^Ac^ ± 1.61
	360	2.68 ^Aa^ ± 0.13	65.14 ^Ac^ ± 6.78	2.14 ^Aa^ ± 0.03	40.93 ^Cd^ ± 2.52	2.70 ^Aa^ ± 0.21	51.69 ^Bd^ ± 1.76	2.52 ^Aa^ ± 0.08	39.87 ^Cd^ ± 0.49
Post-gastrointestinal	28	1.73 ^Ac^ ± 0.003	21.99 ^Bd^ ± 0.07	1.66 ^Ad^ ± 0.07	20.45 ^Bd^ ± 2.05	1.71 ^Ab^ ± 0.005	23.74 ^Bd^ ± 0.37	1.64 ^Ac^ ± 0.10	32.00 ^Ac^ ± 2.83
	90	1.80 ^Bc^ ± 0.01	53.93 ^Ab^ ± 0.81	2.00 ^Ac^ ± 0.02	28.50 ^Cd^ ± 5.80	1.84 ^ABb^ ± 0.06	35.04 ^CDc^ ± 4.20	1.90 ^ABc^ ± 0.05	39.82 ^Dc^ ± 0.77
	180	2.13 ^Ca^ ± 0.01	86.28 ^Ba^ ± 1.01	3.11 ^Ab^ ± 0.01	85.81 ^Ba^ ± 0.36	2.88 ^Ba^ ± 0.06	95.94 ^Aa^ ± 4.05	2.7 ^Bb^ ± 0.03	94.572 ^Aa^ ± 1.67
	270	2.11 ^Aa^ ± 0.23	82.875 ^Ba^ ± 0.36	1.94 ^Ac^ ± 0.24	78.49 ^Cb^ ± 1.216	2.69 ^Aa^ ± 0.26	75.982 ^Bb^ ±1.87	2.21 ^Ab^ ± 0.28	84.42 ^Ab^ ± 2.72
	360	2.31 ^Ca^ ± 0.004	44.88 ^Bc^ ± 1.96	3.44 ^Aa^ ± 0.02	53.63 ^Ac^ ± 0.99	3.03 ^Ba^ ± 0.04	31.09 ^Ccd^ ± 2.43	3.61 ^Aa^ ± 0.45	44.41 ^Bc^ ± 4.17
After simulated adsorption	28	0.63 ^ABc^ ± 0.07	35.00 ^Bb^ ± 2.83	0.54 ^Bc^ ± 0.03	37.438 ^Bb^ ± 3.75	0.60 ^B^ ± 0.01	40.21 ^Ab^ ± 0.56	0.76 ^Ac^ ±0.02	37.91 ^Bb^ ± 2.49
	90	1.27 ^Ca^ ± 0.02	33.92 ^Bb^ ± 1.67	1.60 ^Ba^ ± 0.01	42.32 ^Ab^ ± 5.43	1.78 ^A^ ± 0.01	38.28 ^Ab^ ± 2.96	1.72 ^Aa^ ± 0.02	42.33 ^Ab^ ± 0.14
	180	0.98 ^Bb^ ± 0.09	73.37 ^Ba^ ± 0.95	1.44 ^Aa^ ± 0.07	55.19 ^Da^ ± 1.09	1.40 ^A^ ± 0.03	69.17 ^Ca^ ± 1.16	1.59 ^Aab^ ± 0.07	81.05 ^Aa^ ± 1.48
	270	1.27 ^Aa^ ± 0.02	25.76 ^Bb^ ± 4.79	1.25 ^Aab^ ± 0.08	41.53 ^Ab^ ± 0.10	1.39 ^A^ ± 0.10	42.40 ^Ab^ ± 1.49	1.32 ^Ab^ ± 0.05	39.80 ^Ab^ ± 0.50
	360	0.82 ^Ac^ ± 0.04	30.44 ^Ab^ ± 2.78	1.02 ^Ab^ ± 0.11	20.74 ^Bc^ ± 3.21	0.64 ^A^ ± 0.24	11.35 ^Cc^ ± 0.158	0.75 ^Ac^ ± 0.12	20.41 ^Bc^ ± 2.45

WSF, water-soluble fraction; C, control sample; LOCK, sample inoculated with *Lactobacillus rhamnosus* LOCK900; BAUER, sample inoculated with *Lactobacillus acidophilus* Bauer Ł0938; BB12, sample inoculated with *Bifidobacterium animalis* ssp. *lactis* BB-12. All assays were performed in three replicates, and the results are presented as mean ± standard deviation. ^A–D^ Within the different treatment (verse), means followed by the common letter do not differ significantly (*p* > 0.05).^a–e^ Within the different ageing time(column), means followed by the common letter do not differ significantly (*p* > 0.05).

**Table 4:** Dipeptidyl peptidase IV inhibiting activity (DPP-IVi; %) and peptides concentration (PC, mg/mL) of SSF protein hydrolysates during in vitro digestion of dry-cured pork loins.

Time (Day)		Dipeptidyl-Peptidase-IV-Inhibiting Activity (%)							
		C		LOCK		BAUER		BB12	
		PC (mg/mL)	DPP-IVi(%)	PC (mg/mL)	DPP-IVi(%)	PC (mg/mL)	DPP-IVi(%)	PC (mg/mL)	DPP-IVi(%)
Post-gastric	28	1.31 ^Ab^ ± 0.09	75.88 ^Ba^ ± 0.80	0.92 ^Bb^ ± 0.03	82.79 ^Aa^ ± 4.86	0.76 ^ABb^ ± 0.04	82.80 ^Aa^ ± 0.63	1.09 ^ABb^ ± 0.14	80.31 ^Aa^ ± 0.38
	90	0.75 ^ABc^ ± 0.01	75.51 ^Aa^ ± 0.50	0.64 ^Bb^ ± 0.04	65.93 ^Bb^ ± 3.31	0.62 ^Bb^ ± 0.04	80.28 ^Aa^ ± 1.18	0.83 ^Ac^ ± 0.04	69.69 ^Bb^ ± 0.47
	180	1.65 ^Aa^ ± 0.15	73.10 ^Ba^ ± 1.14	1.66 ^Aa^ ± 0.20	79.51 ^Aa^ ± 0.24	0.72 ^Bb^ ± 0.18	63.97 ^Cb^ ± 1.80	1.65 ^Aab^ ± 0.11	75.74 ^Bab^ ± 2.12
	270	0.74 ^Bc^ ± 0.01	64.76 ^Bb^ ± 1.98	0.64 ^Cb^ ± 0.01	77.35 ^Aa^ ± 2.03	0.82 ^Ab^ ± 0.03	78.89 ^Aa^ ± 1.73	0.75 ^Bc^ ± 0.02	79.15 ^Aa^ ± 1.26
	360	1.79 ^Aa^ ± 0.17	36.25 ^Cc^ ± 2.49	1.91 ^Aa^ ± 0.22	62.88 ^Ab^ ±4.64	1.51 ^Aa^ ± 0.16	34.30 ^Cc^ ± 3.76	1.54 ^Aa^ ± 0.16	48.79 ^Bc^ ± 2.34
Post- gastrointestinal	28	1.42 ^Ac^ ± 0.01	77.41 ^Ab^ ± 4.13	1.43 ^Ab^ ± 0.03	57.52 ^Cc^ ± 2.02	1.37 ^Abd^ ± 0.003	63.83 ^Bc^ ± 0.31	1.46 ^Ac^ ± 0.003	65.85 ^Bc^ ± 0.30
	90	1.38 ^Bc^ ± 0.02	81.93 ^Ab^ ± 6.16	1.53 ^Ab^ ± 0.01	59.48 ^Bc^ ± 2.94	1.08 ^Dd^ ± 0.01	51.17 ^Cd^ ±1.13	1.31 ^Cd^ ± 0.01	59.71 ^Bc^ ± 2.25
	180	1.96 ^Ab^ ± 0.06	92.73 ^Aa^ ± 6.25	2.02 ^Aab^ ± 0.31	95.00 ^Aa^ ± 1.36	1.89 ^Ab^ ± 0.02	95.82 ^Aa^ ± 0.22	1.93 ^Ab^ ± 0.02	93.01 ^Aa^ ± 2.23
	270	1.16 ^Ac^ ± 0.26	82.36 ^Bb^ ± 0.17	1.76 ^Ab^ ± 0.22	82.45 ^Bb^ ± 0.70	1.60 ^Ac^ ± 0.13	89.50 ^Ab^ ± 0.61	1.60 ^Ac^ ± 0.13	83.17 ^Bb^ ± 0.68
	360	3.02 ^Aa^ ± 0.09	39.35 ^Bc^ ± 2.17	2.73 ^Aa^ ± 0.62	42.77 ^Ad^ ± 3.44	2.60 ^Aa^ ± 0.16	29.54 ^Ce^ ± 0.72	2.60 ^Aa^ ± 0.16	28.06 ^Cd^ ± 3.78
After simulated adsorption	28	0.25 ^Ac^ ± 0.04	68.33 ^Aa^ ± 0.30	0.24 ^Ad^ ± 0.003	61.72 ^Bb^ ± 0.81	0.28 ^Ac^ ± 0.05	65.05 ^ABa^ ± 2.30	0.25 ^Ac^ ± 0.05	57.68 ^Cb^ ± 3.04
	90	0.78 ^Aa^ ± 0.01	26.47 ^Dd^ ± 1.05	0.61 ^BCb^ ± 0.04	47.79 ^Cd^ ± 3.08	0.56 ^Cb^ ± 0.003	51.54 ^Bb^ ± 0.47	0.68 ^Ba^ ± 0.003	55.38 ^Ab^ ± 1.07
	180	0.76 ^Aa^ ± 0.08	66.00 ^Bb^ ± 0.70	0.60 ^Ac^ ± 0.02	78.10 ^Aa^ ± 0.05	0.72 ^Aa^ ± 0.008	65.63 ^Ba^ ± 2.50	0.68 ^Aa^ ± 0.008	75.45 ^Aa^ ± 2.97
	270	0.88 ^Aa^ ± 0.01	44.19 ^Cc^ ± 1.75	0.67 ^Bab^ ± 0.07	53.75 ^Ac^ ± 0.27	0.82 ^Aa^ ± 0.07	39.27 ^Dc^ ± 2.42	0.67 ^Ba^ ± 0.07	47.42 ^Bc^ ± 1.18
	360	0.50 ^Bb^ ± 0.04	17.50 ^Be^ ± 2.45	0.86 ^Aa^ ± 0.05	17.61 ^Be^ ± 0.01	0.51 ^Bb^ ± 0.05	20.68 ^Bd^ ± 2.33	0.43 ^Bb^ ± 0.05	27.53 ^Ad^ ± 0.01

SSF, salt-soluble fraction; C, control sample; LOCK, sample inoculated with *Lactobacillus rhamnosus* LOCK900; BAUER, sample inoculated with *Lactobacillus acidophilus* Bauer Ł0938; BB12, sample inoculated with *Bifidobacterium animalis* ssp. *lactis* BB-12. All assays were performed in three replicates, and the results are presented as mean ± standard deviation.^A–D^ Within the different treatment (verse), means followed by the commonletter do not differ significantly (*p* > 0.05).^a–e^ Within the different ageing time(column), means followed by the common letter do not differ significantly (*p* > 0.05).

**Table 5:** List of the peptide sequences with the highest potential for DPP-IVi activity (A parameter higher than 1) after the SGID.

	Peptide	Mass	Protein	C ^1^	LOCK	BB12	BAUER	A Parameter ^2^	Hydrophobicity ^3^	Toxicity ^4^	Allergenicity Probability ^5^	Probability of Being Cell Penetrating ^6^	DPP-IViPredictor ^7^
WSF	VATPPPPPPPK	1096.62	Stress induced phosphoprotein 1 (I3LNG8)			**61.82**		1.2727	−0.09	0	0	0.308375	414.6, DPPIV
	GEAGPAGPAGPAGPR	1260.62	Collagen type I alpha 2 chain (F1SFA7)		77.72	**83.14**		1.2000	−0.06	0	probably allergen	0.230702	284.93, non-DPPIV
	GDRGEAGPAGPAGPAGPR	1588.77	Collagen type I alpha 2 chain (F1SFA7)			**64**		1.1111	−0.18	0	probably allergen	0.272657	261.35, non-DPPIV
	VPVPLPK	748.484	Phosphatase and actin regulator (F1S716)	46.01	46.49	**40.93**	37.1	1.0000	0.04	0	0	0.469216	383.83, DPPIV
	VILPGPAPWG	1005.56	PDZ and LIM domain protein 3 (I3L9B6)	35.71	45.95	**51.76**	43.16	1.0000	0.25	0	0	0.179825	357.44, DPPIV
	NWRPPQPI	1006.53	Carbonicanhydrase 3 (Q5S1S4)	35.83	40.39	35.24	36.74	1.0000	0.13	0	probably allergen	0.235725	398.71, DPPIV
	LILPVGPAGGNQ	1134.64	Protein-L-isoaspartate(D-aspartate) O-methyltransferase (P80895)		38.83	49.2	47.93	1.0000	0.13	0	probably allergen	0.166227	307.36, DPPIV
	VILPGPAPW	948.543	PDZ and LIM domain protein 3 (I3L9B6)		36.97	43.85		1.0000	0.26	0	0	0.190652	381.0, DPPIV
	MLPSLPLL	882.524	Basonuclin 1 (F1RIB5)		41.88	46.2		1.0000	0.25	0	0	0.311738	361.29, DPPIV
	HFFNPVPL	969.507	Hydroxyacyl-coenzyme A dehydrogenase, mitochondrial (P00348)		33.38	33.73		1.0000	0.14	0	0	0.0810493	379.57, DPPIV
	LPLVPVPSPGPPAPL	1449.86	V-set and immunoglobulin domain containing 10-like (F1RP94)		32.81	31.41		1.0000	0.16	0	0	0.235179	373.36, DPPIV
	VPIPVPLPM	961.567	Aldehyde dehydrogenase 6 family member A1 (F1S3H1)		43.19	51.59		1.0000	0.26	0	0	0.150306	425.0, DPPIV
	PQNVILPGPAPWG	1344.71	PDZ and LIM domain protein 3 (I3L9B6)			60.9		1.0000	0.09	0	0	0.182412	359.42, DPPIV
	APPPPAEVH	913.465	Troponin T, fast skeletal muscle (Q75NG9)				31.65	1.0000	−0.03	0	probably allergen	0.121088	401.0, DPPIV
	GLPPPGLT	750.427	Uncharacterized protein (F1SFN5)		40.66			1.0000	0.12	0	probably allergen	0.242026	374.14, DPPIV
	PLALAGPPPP	928.538	Androgen receptor (Q9GKL7)			36.81		1.0000	0.14	0	0	0.259959	395.89, DPPIV
	EPVPLAHPLP	1068.59	Lactamase beta-like 1 (F1STY0)			33.58		1.0000	0.06	0	0	0.176067	394.89, DPPIV
	PQNVILPGPAPW	1287.69	PDZ and LIM domain protein 3 (I3L9B6)			39.14		1.0000	0.08	0	0	0.188348	376.73, DPPIV
	PVVPPLIPPK	1055.67	Troponin T, slow skeletal muscle (Q75ZZ6)				62.9	1.0000	0.09	0	0	0.24149	388.0, DPPIV
	AVSPGLAGPATK	1067.59	Membrane integral NOTCH2 associated receptor 1 (F1RKQ2)				41.94	1.0000	0.04	0	0	0.300649	259.45, non-DPPIV
SSF	GPAGPAGPAGPR	1003.52	Collagen type I alpha 2 chain (F1SFA7)			56.91		1.3333	−0.05	0	probable allergen	0.26505	302.55, DPPIV
	AGPAGPAGPAGPR	1074.55	Collagen type I alpha 2 chain (F1SFA7)		56.12	58.46		1.3077	−0.03	0	probable allergen	0.288459	295.33, DPPIV
	PAGPAGPAGPR	946.498	Collagen type I alpha 2 chain (F1SFA7)		49.15	66.96		1.1818	−0.07	0	probable allergen	0.279523	315.9, DPPIV
	IPAPPGKP	775.459	Guanine nucleotide exchange factor VAV2 isoform 2 (F1S039)			41.85		1.0000	−0.03	0	0	0.159305	362.0, DPPIV
	APPPPAEV	776.406	Troponin T, fast skeletal muscle (Q75NG9)		44.25			1.0000	0.02	0	probable allergen	0.146887	417.0, DPPIV
	KLPPLPL	776.51	Translocase of outer mitochondrial membrane 40 (F1RM44)		30.16			1.0000	0.04	0	0	0.550444	380.5, DPPIV
	VPLPVPVPI	929.59	Retinoicacidinduced 2 (F1SQQ4)		33.14			1.0000	0.29	0	0	0.188433	412.88, DPPIV
	GPPGPPGKP	802.43	Uncharacterized protein (I3L8B2)			71.03		1.0000	−0.11	0	0	0.204097	363.38, DPPIV
	RIPIIP	707.469	RING-type E3 ubiquitin transferase (F1RFJ1)				30.97	1.0000	0.05	0	probable allergen	0.100824	346.2, DPPIV
	RLPLLP	707.469	Kelch-like family member 4 (F1S1P2)				30.97	1.0000	−0.05	0	probable allergen	0.73246	377.4, DPPIV
	SKRLPLP	809.512	Uncharacterized protein (I3LRP9)				34.66	1.0000	−0.31	0	probable allergen	0.609697	277.17, non-DPPIV
	WVGLPPLPSA	1035.57	Bardet–Biedl syndrome 5 protein homolog (F1S1V8)				46.01	1.0000	0.19	0	0	0.239102	350.78, DPPIV

^1^ Blackened field indicates the presence of a sequence; ^2^ score obtained by BIOPEP-UWM database; ^3^ score obtained byToxinpred; ^4^ score obtained by Toxinpred; ^5^ score obtained by Aller Top 2.0.; ^6^ score obtained by CPPpred; ^7^ score obtained by iDPPIV-SCM (values higher than 294 are considered as positive result, and negative results are represented crossed out).WSF, water-soluble fraction; SSF, salt-soluble fraction; C, control sample; LOCK, sample inoculated with Lactobacillus rhamnosus LOCK900; BAUER, sample inoculated with Lactobacillus acidophilus Bauer Ł0938; BB12, sample inoculated with Bifidobacterium animalis ssp. lactis BB-12); DPP-IVi, Dipeptidyl peptidase IV inhibiting activity.

## Data Availability

Not applicable.

## References

[1] Peighambardoust S.H., Karami Z., Pateiro M., Lorenzo J.M. (2021). A Review on Health-Promoting, Biological, and Functional Aspects of Bioactive Peptides in Food Applications. Biomolecules.

[2] Chai K.F., Voo A.Y.H., Chen W.N. (2020). Bioactive peptides from food fermentation: A comprehensive review of their sources, bioactivities, applications, and future development. Comp. Rev. Food Sci. Food Saf..

[3] Toldrá F., Gallego M., Reig M., Aristoy M.C., Mora L. (2020). Bioactive peptides generated in the processing of dry-cured ham. Food Chem..

[4] Fernández-Tomé S., Hernández-Ledesma B. (2020). Gastrointestinal digestion of food proteins under the effects of released bioactive peptides on digestive health. Mol. Nutr. Food Res..

[5] Toldra F., Gallego M., Reig M., Aristoy M.C., Mora L. (2020). Recent progress in enzymatic release of peptides in foods of animal origin and assessment of bioactivity. J. Agric. Food Chem..

[6] Lafarga T., Hayes M. (2014). Bioactive peptides from meat muscle and by-products: Generation, functionality and application as functional ingredients. Meat Sci..

[7] Yoshikawa M., Fujita H., Matoba N., Takenaka Y., Yamamoto T., Yamauchi R., Tsuruki H., Takahata K. (2000). Bioactive peptides derived from food proteins preventing lifestyle-related diseases. Biofactors.

[8] Cicero A.F., Fogacci F., Colletti A. (2017). Potential role of bioactive peptides in prevention and treatment of chronic diseases: A narrative review. Br. J. Pharmacol..

[9] Chaudhury A., Duvoor C., Reddy Dendi V.S., Kraleti S., Chada A., Ravilla R., Marco A., Shekhawat N.S., Montales M.T., Kuriakose K. (2017). Clinical review of antidiabetic drugs: Implications for type 2 diabetes mellitus management. Front. Endocrinol..

[10] Nongonierma A.B., FitzGerald R.J. (2019). Features of dipeptidyl peptidase IV (DPP-IV) inhibitory peptides from dietary proteins. J. Food Biochem..

[11] Molina I., Toldrá F. (1992). Detection of proteolytic activity in microorganisms isolated from dry-cured ham. J. Food Sci..

[12] Fadda S., Sanz Y., Vignolo G., Aristoy M.C., Oliver G., Toldrá F. (1999). Characterization of muscle sarcoplasmic and myofibrillar protein hydrolysis caused by *Lactobacillus plantarum*. Appl. Environ. Microbiol..

[13] Kęska P., Stadnik J. (2018). Stability of antiradical activity of protein extracts and hydrolysates from dry-cured pork loins with probiotic strains of LAB. Nutrients.

[14] Bradford M.M. (1976). A rapid sensitive method for the quantification of microgram quantities of protein utilising the principle of protein-Dye Binding. Anal. Biochem..

[15] Escudero E., Santandreu M.A., Toldra F. (2010). Characterization of peptides released by in vitro digestion of pork meat. J. Agric. Food Chem..

[16] Adler-Nissen J. (1979). Determination of the degree of hydrolysis of food protein hydrolysates by trinitrobenzenesulfonic acid. J. Agric. Food Chem..

[17] BIOPEP-UWM. https://biochemia.uwm.edu.pl/biopep-uwm/.

[18] ToxinPred. http://crdd.osdd.net/raghava/toxinpred/.

[19] Aller Top. https://www.ddg-pharmfac.net/AllerTOP/.

[20] CPPpred. http://distilldeep.ucd.ie/CPPpred/.

[21] iDPPIV-SCM. http://camt.pythonanywhere.com/iDPPIV-SCM.

[22] Singh H., Singh S., Raghava G.P.S. (2019). Peptide secondary structure prediction using evolutionary information. BioRxiv.

[23] Kurcinski M., Ciemny M.P., Oleniecki T., Kuriata A., Badaczewska-Dawid A.E., Kolinski A., Kmiecik S. (2019). CABS-dock standalone: A toolbox for flexible protein–peptide docking. Bioinformatics.

[24] CABSdock. https://bitbucket.org/lcbio/cabsdock/wiki/Home.

[25] Kęska P., Stadnik J. (2016). Porcine myofibrillar proteins as potential precursors of bioactive peptides–An in silico study. Food Funct..

[26] Kęska P., Stadnik J., Wójciak K.M., Neffe-Skocińska K. (2020). Physico-chemical and proteolytic changes during cold storage of dry-cured pork loins with probiotic strains of LAB. Int. J. Food Sci. Technol..

[27] Kęska P., Stadnik J. (2019). Ageing-time dependent changes of angiotensin I-converting enzyme-inhibiting activity of protein hydrolysates obtained from dry-cured pork loins inoculated with probiotic lactic acid bacteria. Int. J. Pept. Res. Ther..

[28] Ashraf A., Mudgil P., Palakkott A., Iratni R., Gan C.Y., Maqsood S., Ayoub M.A. (2021). Molecular basis of the anti-diabetic properties of camel milk through profiling of its bioactive peptides on dipeptidyl peptidase IV (DPP-IV) and insulin receptor activity. J. Dairy Sci..

[29] Rivero-Pino F., Espejo-Carpio F.J., Guadix E.M. (2020). Production and identification of dipeptidyl peptidase IV (DPP-IV) inhibitory peptides from discarded Sardine pilchardus protein. Food Chem..

[30] Mudgil P., Kilari B.P., Kamal H., Olalere O.A., FitzGerald R.J., Gan C.Y., Maqsood S. (2020). Multifunctional bioactive peptides derived from quinoa protein hydrolysates: Inhibition of α-glucosidase, dipeptidyl peptidase-IV and angiotensin I converting enzymes. J. Cereal Sci..

[31] Lacroix I.M., Li-Chan E.C. (2012). Evaluation of the potential of dietary proteins as precursors of dipeptidyl peptidase (DPP)-IV inhibitors by an in silico approach. J. Funct. Foods.

[32] Lafarga T., O’Connor P., Hayes M. (2014). Identification of novel dipeptidyl peptidase-IV and angiotensin-I-converting enzyme inhibitory peptides from meat proteins using in silico analysis. Peptides.

[33] Singla R.K., Kumar R., Khan S., Kumari K., Garg A. (2019). Natural Products: Potential Source of DPP-IV Inhibitors. Curr. Protein Pept. Sci..

[34] Martini S., Conte A., Tagliazucchi D. (2019). Comparative peptidomic profile and bioactivities of cooked beef, pork, chicken and turkey meat after in vitro gastro-intestinal digestion. J. Proteom..

[35] Marušić N., Aristoy M.C., Toldrá F. (2014). Nutritional pork meat compounds as affected by ham dry-curing. Meat Sci..

[36] Gallego M., Aristoy M.C., Toldrá F. (2014). Dipeptidyl peptidase IV inhibitory peptides generated in Spanish dry-cured ham. Meat Sci..

[37] Nongonierma A.B., FitzGerald R.J. (2013). Dipeptidyl peptidase IV inhibitory properties of a whey protein hydrolysate: Influence of fractionation, stability to simulated gastrointestinal digestion and food–drug interaction. Int. Dairy J..

[38] Huang S.L., Jao C.L., Ho K.P., Hsu K.C. (2012). Dipeptidyl-peptidase IV inhibitory activity of peptides derived from tuna cooking juice hydrolysates. Peptides.

[39] Harnedy P.A., O’Keeffe M.B., FitzGerald R.J. (2015). Purification and identification of dipeptidyl peptidase (DPP) IV inhibitory peptides from the macroalga *Palmaria palmata*. Food Chem..

[40] Vilcacundo R., Martínez-Villaluenga C., Hernández-Ledesma B. (2017). Release of dipeptidyl peptidase IV, α-amylase and α-glucosidase inhibitory peptides from quinoa (Chenopodium quinoa Willd.) during in vitro simulated gastrointestinal digestion. J. Funct. Foods.

[41] Goodman R.E. (2014). Biosafety: Evaluation and regulation ofgenetically modified (GM) crops in the United States. J. Huazhong Agric. Univ..

[42] Hayes M., Rougé P., Barre A., Herouet-Guicheney C., Roggen E.L. (2015). In silico tools for exploring potential human allergy to proteins. Drug Discov. Today Dis. Models.

[43] Derakhshankhah H., Jafari S. (2018). Cell penetrating peptides: A concise review with emphasis on biomedical applications. Biomed. Pharmacother..

[44] Kim G.C., Cheon D.H., Lee Y. (2021). Challenge to overcome current limitations of cell-penetrating peptides. Biochim. Biophys. Acta (BBA)-Proteins Proteom..

[45] Gupta S., Kapoor P., Chaudhary K., Gautam A., Kumar R., Raghava G.P., Open Source Drug Discovery Consortium (2013). In silico approach for predicting toxicity of peptides and proteins. PLoS ONE.

[46] Lacroix I.M., Li-Chan E.C. (2012). Dipeptidyl peptidase-IV inhibitory activity of dairy protein hydrolysates. Int. Dairy J..

[47] Power O., Nongonierma A.B., Jakeman P., FitzGerald R.J. (2014). Food protein hydrolysates as a source of dipeptidyl peptidase IV inhibitory peptides for the management of type 2 diabetes. Proc. Nutr. Soc..

[48] Boots J.-W.P. (2012). Protein hydrolysate enriched in peptides inhibiting DPP-IV and their use. U.S. Patent.

[49] Engel M., Hoffmann T., Wagner L., Wermann M., Heiser U., Kiefersauer R., Huber R., Bode W., Demuth H.U., Brandstetter H. (2003). The crystal structure of dipeptidyl peptidase IV (CD26) reveals its functional regulation and enzymatic mechanism. Proc. Natl. Acad. Sci. USA.

[50] Raveschot C., Cudennec B., Coutte F., Flahaut C., Fremont M., Drider D., Dhulster P. (2018). Production of bioactive peptides by Lactobacillus species: From gene to application. Front. Microbiol..

[51] Jensen M.P., Ardö Y. (2010). Variation in aminopeptidase and aminotransferase activities of six cheese related *Lactobacillus helveticus* strains. Int. Dairy J..

